# Effect of Forest Structural Change on Carbon Storage in a Coastal *Metasequoia glyptostroboides* Stand

**DOI:** 10.1155/2013/830509

**Published:** 2013-09-25

**Authors:** Xiangrong Cheng, Mukui Yu, Tonggui Wu

**Affiliations:** Institute of Subtropical Forestry, Chinese Academy of Forestry, Fuyang, Zhejiang 311400, China

## Abstract

Forest structural change affects the forest's growth and the carbon storage. Two treatments, thinning (30% thinning intensity) and underplanting plus thinning, are being implemented in a coastal *Metasequoia glyptostroboides* forest shelterbelt in Eastern China. The vegetation carbon storage significantly increased in the underplanted and thinned treatments compared with that in the unthinned treatment (*P* < 0.05). The soil and litterfall carbon storage in the underplanted treatment were significantly higher than those in the unthinned treatment (*P* < 0.05). The total forest ecosystem carbon storage in the underplanted and thinned treatments increased by 35.3% and 26.3%, respectively, compared with that in the unthinned treatment, an increase that mainly came from the growth of vegetation aboveground. Total ecosystem carbon storage showed no significant difference between the underplanted and thinned treatments (*P* > 0.05). The soil light fraction organic carbon (LFOC) was significantly higher at the 0–15 cm soil layer in the thinned and underplanted stands compared with that in the unthinned stand (*P* < 0.05). The soil respiration of the underplanted treatment was significantly higher than that of the unthinned treatment only in July (*P* < 0.05). This study concludes that 30% thinning and underplanting after thinning could be more favorable to carbon sequestration for *M. glyptostroboides* plantations in the coastal areas of Eastern China.

## 1. Introduction

Forests play an important role in the global carbon cycle; more than 80% of all terrestrial aboveground carbon and more than 70% of all soil organic carbon are stored in forest ecosystems [1]. Nearly 3 billion tons carbon of anthropogenic carbon every year (3 Pg C year^−1^) was removed through net growth [2]. Many studies have reported that forest management practices might affect carbon dynamics in forest ecosystems [3–5]. Therefore, much attention has been given to forest management to improve carbon sinks and mitigate global climate change [1, 2], for example, increase of forested land area through reforestation, increase of the carbon density of existing forests at stand and landscape scales, and reduction of carbon emissions from deforestation and degradation.

Changes of forest structure will lead to different forest environments, which will further influence forest growth and total carbon stocks [1, 6]. Thinning and underplanting are two important silvicultural strategies to change the monoculture stand structure, and they are widely applied in plantation management [4, 7]. Most research on forest thinning has been performed to identify silvicultural practices to produce optimal stand growth. In recent years, some studies have focused on the implications of thinning for carbon [7–9]. Previous studies reported that moderate thinning treatment could be more effective to conserve vegetation carbon storage [10, 11]. However, less experimental evidence was available for the effect of thinning on the carbon pool in the mineral soil; soil carbon storage and soil respiration were found to vary in a few thinning studies [1]. Soil carbon might (temporarily) be enhanced due to increased litter input, but a change in microclimate could also lead to increased decomposition. A decrease in soil respiration with increasing stand density could be expected, as soil temperatures often decreased with increasing density. Meanwhile, the change of soil labile carbon fractions (e.g., light fraction organic carbon, LFOC) was found to influence soil carbon stability, which was greatly affected by the forest environment [12]. However, the long-term effects of thinning on belowground carbon stocks and soil respiration of forests are poorly understood [10].

Uneven-aged forest management has increased the significance of nature conservation, ecosystem services, forest resilience, and stability in a changing climate. There has been growing interest in uneven-aged silviculture, and it has become increasingly important in the last few decades particularly in relation to carbon management [13]. The underplanting of trees beneath pure, even-aged plantations has been applied in various parts of the world; for example, broadleaved tree species were planted under spruce (*Picea abies*) plantations in Europe [14]. However, it is less clear that underplanting in natural or planted forest how to affect forest carbon storage and CO_2_ flux. It was reported that uneven-aged Norway spruce and Scots pine (*Pinus sylvestris*) stands had higher timber benefits than even-aged stands in Central Finland [15]. Chatterjee et al. [16] noted that total ecosystem carbon storage of ponderosa pine (*Pinus ponderosa*) forest showed no significant difference between a 46-year-old even-aged stand (164 Mg C ha^−1^) and a 110-year-old uneven-aged stand (170 Mg C ha^−1^) in Wyoming.

*Metasequoia glyptostroboides* is deciduous tree species, one of the main tree species of shelterbelt in the eastern coastal area of China [17]. However, ecological function of the plantation is seriously affected by the monoculture patterns due to the simple stand structure and the relatively high stand density. The protective effect of shelterbelt may differ greatly due to the different tree species composition and vertical stand structure [18]. Some stand structural change measures (e.g., thinning and underplanting) were implemented in the study area, but those studies mainly focused on improving the stand environment and the forest protective effect [19]. Little is known about the effect of structural changes of monoculture on aboveground and underground carbon storage and soil respiration. The objectives of this study were to (1) investigate the difference of soil, litterfall, and overstory and understory vegetation carbon storage among three treatments (unthinning, thinning, and underplanting plus thinning) in an *M. glyptostroboides* stand; (2) determine the effect of forest structural change on total forest system carbon storage; and (3) determine the response of soil LFOC and CO_2_ fluxes to forest structural change. We hypothesized that (i) forest structural change would lead to great variation on above- and belowground carbon storage and its allocation in *M. glyptostroboides* stand and (ii) that sole thinning did not reduce total system carbon storage, while underplanting plus thinning treatment would increase total system carbon storage, because underplanting enhances above- and belowground carbon storage.

## 2. Experimental Section

### 2.1. Site Description

The study was conducted at the *M. glyptostroboides* coastal forest shelterbelt in Pudong District of Shanghai, China (121°52′43′′E, 31°56′50′′N; mean altitude 2-3 m). The shelterbelt was established in 1986 and is approximately 50 m wide and 23 km long. The initial tree density was 1400 trees ha^−1^; due to sapling death and wind damage, the current tree density is approximately 900 trees ha^−1^. In the study area, the mean annual temperature is 15.6°C and the mean annual precipitation is 1137.1 mm, most of which falls from June to September. The annual sunshine time is 1993.4 h, and the frost-free time is 223 d, belonging to the northern subtropical marine monsoon climate. According to FAO soil classification [20], the soil type in the experimental area is solonchak with 14.6–17.5% clay, 60.2–72.5% silt, and 12.7–22.5% sand; pH is 7.6.

### 2.2. Thinning and Underplanting Treatments

In 2000, the 5 km* M. glyptostroboides* forest shelterbelt was thinned with a 30% thinning intensity (small trees and wind damaged trees were cut), which included three 1.5–2 km long shelterbelt. In each thinned section, approximately half of the thinned stand was underplanted with *Broussonetia papyrifera *(a widely distributed deciduous tree species in China) to establish the uneven-aged forest. The underplanted tree density was approximately 1500 trees ha^−1^. Completely randomized design with a single factor was used in this experiment. One 25 m × 25 m sample plot was randomly established in each thinned, underplanted, and unthinned *M. glyptostroboides* stands on July, 2000. The selected experimental area was in the middle of the whole forest shelterbelt, because stand characteristics (e.g., tree growth and density) and site properties (e.g., soil texture and pH) were similar in this area before the experiment. Tree height, diameter at breast height (DBH), and density of all tree size classes were measured in all plots at the beginning of the experiment. Before the treatment, for thinned, underplanted, and unthinned plots, mean tree height was 10.6 m, 10.3 m, and 9.7 m, respectively; mean DBH was 12.1 cm, 12.5 cm, and 11.9 cm, respectively; mean density was 964 trees ha^−1^, 948 trees ha^−1^, and 986 trees ha^−1^, respectively. Stand characteristics in pretreatment showed no significant differences among the three treatments (*P* > 0.05). Current characteristics of overstory tree and understory vegetation in these sample plots were investigated in 2009 (Table 1).

### 2.3. Tree Biomass

The diameter at breast height (DBH), tree height (H), and tree density was investigated in all the plots. For biomass dry weight, functions developed by Wang [21] in nearby area, with diameter and height as explanatory variables, were used. The aboveground and underground biomass of the *M. glyptostroboides* stands were estimated by allometric functions (*y*_aboveground_ = 0.153(DBH^2^H)^0.857^, *y*_underground_ = 0.045(DBH^2^H)^0.853^). The average carbon content of the aboveground and underground of the *M. glyptostroboides* trees was 0.46 g g^−1^ and 0.41 g g^−1^, respectively [21]. Carbon storage at the plot level was determined by summing up the carbon in the aboveground/underground for all trees and underground components.

### 2.4. Understory Vegetation

Understory vegetation biomass was estimated from destructive harvests. The DBH, H, and tree density of the understory woody plants (including trees and shrubs) were investigated in all the plots. The understory woody plant was mainly *Pterocarya stenoptera* in the thinned and unthinned treatments, and it was* B. papyrifera* in the underplanted treatment. According to the average DBH and H, five representative sample trees were selected in each plot and the aboveground/underground components were harvested. The roots of three of the five sampled trees were excavated using a hand power puller and manually digging combination approach. Nine subplots of 1 m × 1 m were randomly set in each plot, and herbs (mainly *Phytolacca acinosa *and *Oxalis corniculata*) within each subplot were harvested. The subsamples of the understory woody plants and herbs were dried at 70°C until a constant mass was reached and weighed, and the aboveground and underground dry biomass were calculated. The carbon content was set at 0.45 g g^−1^ for understory woody plants and at 0.34 g g^−1^ for herbs [22]. Then, the understory woody plant and herb carbon storage were determined according to the plant dry biomass, carbon content, and plant density.

### 2.5. Litterfall Collection

Five 1 m × 1 m subplots were randomly set within each plot, and all the litterfall of each subplot on the ground was collected. Dry biomass was obtained by drying the litterfall samples at 70°C until a constant mass was reached. The carbon content of the litterfall was analyzed; it was 0.43 g g^−1^, 0.43 g g^−1^, and 0.44 g g^−1^ for thinned, unthinned, and underplanted treatments, respectively. Then, the litterfall carbon storage per unit area was estimated according to the dry mass and carbon content of the litterfall in the subplot.

### 2.6. Soil Sampling

In the six sample plots, a soil drill (Ø 4 cm) was utilized to take soil samples randomly from five points with depths of 0–15 cm, 15–30 cm, 30–50 cm, and 50–100 cm. The samples were bulked, air-dried for 1-2 months, and sieved through a 2 mm mesh, and the soil organic carbon concentrations of these samples were analyzed. The plant, litterfall, and soil carbon contents were determined according to the potassium dichromate oxidation-external heating method [23]. Soil organic carbon (SOC, t C ha^−1^) storage was calculated with the following formula:

(1) $$\begin{array}{c} \text{SOC}=\overset{n}{\underset{i}{\sum }}C_{i}\times H_{i}\times B_{i}\times 10, \end{array}$$

where *C*_*i*_ is the soil organic carbon content (g kg^−1^), *H*_*i*_ is the depth of each soil layer (cm), and *B*_*i*_ is soil bulk density of each soil layer (g kg^−1^) [24]. Due to the low stone content in the soil in the study area, stone content was neglected in the soil organic carbon storage calculation.

### 2.7. Soil Light Fraction Organic Carbon (LFOC)

LFOC was separated by flotation on a NaI solution with a density of 1.70 g cm^−3^ using a modification of the method described by Janzen et al. [25]. Three 10 g subsamples of each soil (<2 mm) were weighed in a 100 mL plastic centrifuge tube, and 50 mL of NaI liquid solution was added to the tube. The tubes were shaken on a shaker for 30 min and then centrifuged at 1,000 rpm for 10 min. The floating light fraction was immediately removed and poured into a plastic bottle. This process was repeated three times, and the supernatant material was poured into the previous plastic bottle. The light fraction was collected through a membrane filter (0.45 *μ*m) in a Büchner funnel; it was washed three times with 0.01 mol L^−1^ CaCl_2_ to remove excess NaI. The material was washed again three times with deionized water, after which it was dried at 60°C for 24 h and weighed and finely ground for the determination of organic carbon.

### 2.8. Soil Respiration, Temperature, and Moisture

Soil respiration rates were measured from July 2009 to April 2010 using a soil respiration chamber (LI-6400-09; LiCor, Lincoln, Nebraska, USA) connected to a portable photosynthesis system (LI-6400). Six PVC collars (10 cm high, 8 cm inside diameter) were randomly inserted into soil of each plot at a depth of 5 cm. The ground vegetation was firmly removed before measurement to minimize the disturbance. Altogether, 36 spots were measured from 9:00 A.M. to 11:00 A.M. in mid-July, mid-October, mid-January, and mid-April. On each occasion, all spots were measured two to three times depending on the variation in the results. The mean value from these readings for each spot was used for further analysis.

Soil temperature at a depth of 5 cm was measured simultaneously with the CO_2_ flux measurement with a thermocouple as an integrated part of the CO_2_ monitoring system. Soil moisture was measured as volumetric content (%) directly after the CO_2_ efflux measurement in the middle of the sample plot at a 5 cm depth (moisture meter type HH2, Delta-T Devices Ltd.).

### 2.9. Statistical Analysis

The experimental data were analyzed and processed using Excel 2003 and SPSS16.0 (SPSS Inc., Chicago, Illinois, USA). The differences of soil, litterfall, overstory and understory vegetation carbon storage, LFOC, and soil respiration between the thinned, underplanted, and unthinned stands were examined by one-way ANOVA and Duncan multiple comparisons (*α* = 0.05). Pearson correlation analysis was used to evaluate the relationship between soil respiration and soil temperature and soil water content.

## 3. Results

### 3.1. Above- and Belowground Carbon

The vegetation carbon storage of aboveground and underground in the underplanted and thinned treatments was significantly higher than that of the unthinned treatment (*P* < 0.05) (Table 2). Although the vegetation carbon storage of the underplanted treatment was slightly higher than that of the thinned treatment, there was no significant difference between them (*P* > 0.05). The understory woody plants and herbs of the underplanted treatment contributed to 4.7% and 0.2% of the total vegetation carbon storage, respectively. These values were 0.8% and 0.7% for the thinned and 0.3% and 0.1% for the unthinned treatments, respectively. These results suggest that the underplanted trees beneath the thinned *M. glyptostroboides* stand could significantly increase the understory vegetation carbon storage (*P* < 0.05). Moreover, the litterfall and SOC storage of the underplanted treatment were significantly higher than those of the unthinned treatments (*P* < 0.05). There was no significant difference in litterfall and SOC storage between the thinned and unthinned treatments (*P* > 0.05). The order of the total forest ecosystem carbon storage of the three treatments was underplanted > thinned > unthinned treatment, but the difference was not significant between the underplanted and thinned treatments (*P* > 0.05). We need to emphasize that this result did not include the thinned carbon loss; in fact, they were very small (<10 t C ha^−1^) compared with the current total forest ecosystem carbon storage for the unthinned and underplanted treatments.

**Figure 1:**
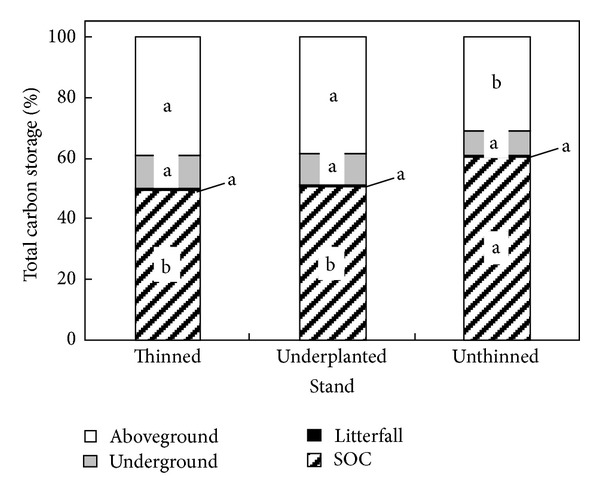
The percentages of vegetation aboveground/underground, litterfall, and soil carbon storage accounting for total stand carbon storage in the thinned, underplanted, and unthinned treatments; different letters in the same component indicate there are significant differences among the three treatments (5% level, Duncan's test).

The SOC storage of the underplanted and thinned treatments accounted for 49.5% and 50.5% of total ecosystem carbon storage, respectively, and the percentage was 60.2% for the unthinned treatment (Figure 1). The vegetation aboveground carbon storage of the underplanted and thinned treatments accounted for 39.2% and 38.2% of total ecosystem carbon storage, respectively, which was 30.6% for the unthinned treatment. Litterfall carbon storage comprised approximately 0.9% of the total ecosystem carbon storage in the three treatments. The percentages of the vegetation underground carbon storage accounting for the total ecosystem carbon storage were similar in the three treatments, and those of the underplanted and thinned treatments were slightly higher than those of the unthinned treatment. This result indicates that the increase of carbon storage in the underplanted and thinned treatments mainly originated from the growth of the vegetation aboveground.

### 3.2. Soil Carbon Storage

**Figure 2:**
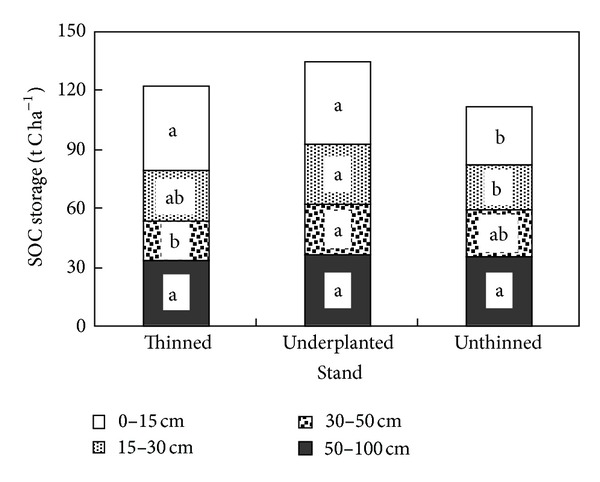
SOC storage (t C ha^−1^) in the 0–15 cm, 15–30 cm, 30–50 cm, and 50–100 cm soil layers in the thinned, underplanted, and unthinned treatments; different letters in the same soil layer indicate there are significant differences among the three treatments (5% level, Duncan's test).

The size of SOC storage presented great difference among the three treatments at different soil layers (Figure 2). The SOC storage in 0–15 cm soil layer was significantly higher in the thinned and underplanted treatments than that in the unthinned treatment (*P* < 0.05). In 15–30 cm and 30–50 cm soil layers, the values of SOC storage in the underplanted treatment were also the highest among the three treatments. SOC storage in 50–100 cm soil layer showed no significant difference among the three treatments (*P* > 0.05). The SOC of the 0–15 cm soil layer of the three treatments was the highest among all the sampled layers, comprising 35.9% of the total carbon storage of the entire profile (0–100 cm) of the thinned stands, whereas those of the underplanted and unthinned treatments were 31.5% and 26.1%, respectively. This indicated that the SOC storage of the surface soil was significantly increased in both the thinned and underplanted stands. The total SOC (0–100 cm soil layer) in the underplanted and thinned stands increased by 23.6 t C ha^−1^ and 11.2 t C ha^−1^, respectively, compared with that of the unthinned stands.

### 3.3. Soil Light Fraction Organic Carbon (LFOC)

The average LFOC concentrations decreased with soil depth in the three stands and were significantly higher at the 0–15 cm and 15–30 cm depths compared with the other soil depths (Table 3). In the 0–15 cm layer, the concentrations of LFOC in the thinned and underplanted stands were significantly higher than those of the unthinned stand, with no significant differences detected between the thinned and underplanted stands. The LFOC concentrations showed no significant differences at the 15–30 cm, 30–50 cm, and 50–100 cm depths among the three stands. The difference of LFOC/SOC through the soil profile was not significant among the three stands.

### 3.4. Soil Respiration

**Figure 3:**
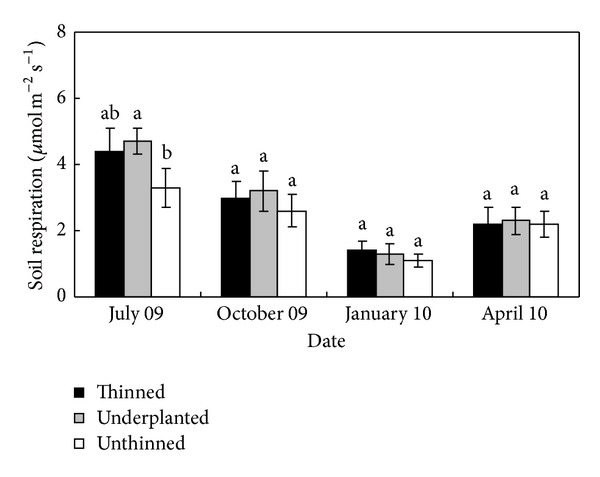
Seasonal change of soil respiration (*μ*mol m^−2^ s^−1^) in the thinned, underplanted, and unthinned treatments; different letters in the same date indicate there are significant differences among the three treatments (5% level, Duncan's test).

The patterns of seasonal change of soil respiration were similar for the thinned, underplanted, and unthinned treatments during the study period (Figure 3). The highest soil respiration rates of the three treatments occurred in July, followed by October and April, with the lowest value in January. The soil respiration of the underplanted treatment was significantly higher than that of the unthinned treatment in July, and no significant differences were noted between the thinned and the underplanted treatment. The soil respirations showed no significant differences among the three treatments in October, January, and April. Soil respiration of the three treatments had a positive correlation with soil temperature and soil moisture with time (Table 4).

## 4. Discussion

### 4.1. Response of Carbon Storage to Forest Structural Change

In the current study, we observed more rapid growth rate of the thinned forest and greater above- and belowground carbon accumulation in the thinned and underplanted plots than in the control plots. Ten years after the treatment, higher carbon accumulation of the thinned and underplanted plots was mainly coming from the aboveground faster growth of *M. glyptostroboides* and understory vegetation (especially for underplanted plots). The aboveground carbon (AGC) accumulation rates in the thinned and underplanted plots are 3.69 t C year^−1^and 4.22 t C year^−1^. Our results are comparable to carbon sequestration found in thinned plots in floodplain forests in Australia [10]. Their study showed that higher rates of AGC storage were in thinned stands (3.1–4.1 t C year^−1^) compared to unthinned stands (1.6 t C year^−1^); particularly it was highest in the moderate thinned treatment (560 trees ha^−1^ treatment). Additionally, Vargas et al. [8] also observed that AGC storage in the thinned plots was slightly higher than that in the unthinned plots after five years of treatment. Similar results also were detected by Hoover and Stout [7]. In contrast, Keyser [26] observed that AGC storage decreased with increasing thinning intensity after 35 years of postthinning growth in pure yellow-poplar stands in the Southern Appalachian Mountains. Thinning decreased the number of stems in a forest stand and reduced competition from small-diameter trees, which may have been using belowground resources (e.g., water, nutrients) [27]. At high stand density, individual tree growth is limited by crown size at the time of canopy closure. Higher tissue respiration demands relative to photosynthetic area mean less photosynthate is available for allocation to growth [28]. Moreover, underplanting could increase stand biodiversity [29] and improve the soil quality [30], including soil organic carbon. These results confirmed our hypothesis that changes in stand structure could greatly influence carbon storage.

It was reported that thinning increased canopy openness and enhanced surface temperature, which would speed up the litterfall decomposition and thus reduce the amount of litterfall [1]. Nevertheless, contrary results were obtained in this study, which may be relevant to stand age at the time of thinning treatment, site history, and vegetation type. The reduced canopy density shortly after thinning would promote litterfall decomposition, whereas the growth of the remained trees and understory vegetation would gradually increase the amount of litterfall.

Ten years after the treatment, total ecosystem carbon storage in the thinned (248.66 t C ha^−1^) and underplanted plots (269.35 t C ha^−1^) was significantly higher than that in the unthinned plots (197.32 t C ha^−1^); no significant difference was found between the thinned and underplanted plots. These results suggest the fast recovery potential of *M. glyptostroboides *plantation after thinning. However, many short-term studies have suggested that thinning would reduce the tree density, leading to the decline of the stand productivity [31, 32]. Nevertheless, thinning would improve the light environment within the forest and facilitate the growth of understory vegetation, which would partially remedy the carbon storage removed by the cut trees after thinning [33]. Our results also confirmed that especially underplanting might be a more effective measure to increase forest carbon sink. At present, the long-term effect of thinning on above- and belowground carbon stocks was still poorly understood. Although the literature suggests that carbon is maximized by reducing disturbance events [34]; many studies showed that stand volume (from which the conversion to live tree carbon is relatively straightforward) had small increase after light to moderate levels of thinning [35, 36]. Numerous model simulation studies have examined the potential long-term impacts of alternative management scenarios on stand level carbon stocks [37–39]; however, there is a paucity of data-driven research that specifically addresses the effects of thinning and other stand management activities on long-term changes in carbon storage. Several long-term thinning studies mainly focused on aboveground live tree carbon stocks [7, 9, 10], less involved in belowground carbon stocks [10]. Therefore, more research needs to be done to fully understand the long-term effects of thinning on carbon uptake and storage.

In this study, the current carbon stocks in *M. glyptostroboides *stands with different forest structure were investigated. Therefore, logs removed in the thinning were counted as carbon releases. Depending on the end use, however, these logs removed could either produce carbon offsets if long-lived wood products are produced or could lead to net carbon releases over the next 100 years if short-lived wood products are produced [9, 40]. However, biomass energy facilities can capture and utilize energy from burning wood, which provide a carbon neutral substitute for other fossil fuels [41]. Therefore, these carbon releases could potentially become carbon neutral, which would increase the net carbon storage in thinned and underplanted treatments.

### 4.2. Effect of Forest Structural Change on Soil Carbon Pool

In the current study, the SOC storage in the underplanted and thinned treatments was higher than that in the unthinned treatment, but the underplanted treatment markedly increased C pool compared with the unthinned treatment (*P* < 0.05); no significant difference was found between the thinned treatment and the underplanted treatment or unthinned treatment (*P* > 0.05). This indicated that only moderate thinning did not affect the SOC storage for the *M glyptostroboides* stand and underplanting after thinning had a positive effect to increase soil carbon sequestration. Our results are consistent with the results (five years after thinning) of the thinning experiment in a seasonally dry tropical forest in Mexico by Vargas et al. [8]. Kim et al. [5] also found that thinning did not significantly affect the SOC storage in a *Pinus densiflora*. stand in Korea in a short time (two years after thinning). It was reported that the soil temperature and humidity were increased after thinning, which would accelerate the decomposition of the soil organic matter, leading to reduced SOC storage [42]. In this study, the ground litterfall and stand roots of the thinned and underplanted treatments were significantly higher than those of the unthinned treatment (*P* < 0.05), and the litterfall and roots of the aboveground and underground vegetation were the main source of the soil organic matter, which may be the main reason for the soil carbon storage differences among the thinned, underplanted, and unthinned treatments, although we did not investigate fine root biomass.

The light fraction serves as a readily decomposable substrate for soil microorganisms, which is a short-term reservoir of plant nutrients and the primary fraction for soil carbon formation [43]. Its size is a balance between residue inputs and decomposition [44]. The LFOC of the topsoil (0–15 cm) in the thinned and underplanted stands was significantly higher than that of the unthinned stand, with no significant differences detected between the thinned and underplanted stands. Some studies also found that the LFOC was higher at the soil surface, which was strongly related to root carbon inputs [45] and other organic residues accumulated in this layer. Our results are consistent with the previous findings [12, 46]. In this study, the higher LFOC at the 0–15 cm layer in the thinned and underplanted stands could be related to more root and litterfall carbon inputs (Table 2).

The seasonal patterns of soil respiration in the three treatments were consistent with other studies observing forest ecosystems [47, 48]. Some researchers have found that soil respiration decreased 1–3 years after thinning [49, 50]. The decrease in soil respiration in thinned forests has been attributed to a greater reduction in autotrophic (root) respiration than to an increase in heterotrophic respiration [49]. However, Tian et al. [51] showed that soil respiration increased after thinning (year 1) in Chinese fir plantations, and then decreased, with no difference noted in the 8th year between thinned and unthinned stands. They noted that the initial increase in CO_2_ could be attributed to a combination of root decay, soil disturbance, and increased soil temperature in gaps.

Many researchers have demonstrated that soil respiration temporal changes were mostly explained by soil temperature and soil moisture [52, 53] and soil respiration had a less significant relationship with the two variables at the spatial scale [54]. In this study, the thinned and underplanted treatments had higher soil respiration in July, no significant differences were observed among the three treatments in October, January, and April, which could be related to the higher root and litterfall content in the two treatments. An increase of root biomass could enhance autotrophic respiration, and more litterfall input could stimulate heterotrophic respiration. Moreover, the higher LFOC of the topsoil in the thinned and underplanted stands provided readily decomposable substrate for soil microorganisms and thus increased CO_2_ release.

## 5. Conclusions

Underplanted and thinned treatments markedly changed the spatial distribution of carbon stocks compared with an unthinned treatment in *M. glyptostroboides* plantations. The carbon storage of the vegetation aboveground/underground and the soil and litterfall in the underplanted treatment were significantly higher than those of the unthinned treatment. The vegetation carbon storage of the thinned treatment was also significantly higher than that of the unthinned one, with no significant differences detected in the soil and litterfall carbon storage between the thinned and unthinned treatments. The total forest ecosystem carbon storage was significantly higher in the underplanted and thinned treatments than in the unthinned one, but the difference was not significant between the underplanted and thinned treatments. Only single moderate thinning did not greatly influence the soil C inputs and release, and underplanting after thinning could have a complex effect on soil C pool. Underplanted stand increased SOC storage; on the one hand, it also enhanced soil respiration, although not occurred in the whole growing season. Unfortunately, we lacked information on the carbon balance of the *M. glyptostroboides* plantations. More research is needed on the carbon transfer and balance of thinning management in plantations. However, because these sample plots were localized in the same stand, the experiment was not truly replicated, and it is worthwhile to note that this is a case study on forest structure change and future studies should incorporate other observations in other ecosystems to further support these findings. Even so, the two silvicultural measures (underplanting and thinning) will be retaining much potential to sequestrate carbon in coastal *M. glyptostroboides* plantations. Especially, underplanting in monoculture plantation will be an important way to develop multifunction forest.

## Figures and Tables

**Table 1:** Characteristics of vegetation in thinned, underplanted, and unthinned stands (standard deviation in brackets); DBH is diameter at breast height.

	Overstory tree			Understory woody plant			Herb	
Stands	Height (m)	DBH (cm)	Density (trees ha^−1^)	Height (m)	DBH (cm)	Density (trees ha^−1^)	Height (m)	Coverage (%)
Thinned	19.3 (0.8)	21.8 (1.4)	600 (57)	2.7 (0.7)	4.1 (1.5)	491 (58)	0.8 (0.1)	91.5 (4.9)
Underplanted	19.8 (1.3)	22.1 (2.2)	582 (52)	4.0 (0.3)	4.4 (1.3)	1680 (85)	0.6 (0.1)	59.0 (8.5)
Unthinned	13.9 (0.5)	15.8 (1.1)	865 (53)	2.1 (0.5)	2.8 (0.1)	278 (39)	0.3 (0.1)	12.5 (3.5)

**Table 2:** Carbon storage in the thinned, underplanted, and unthinned treatments (standard deviation in brackets); different letters in the same line indicate there are significant differences among the three treatments (5% level, Duncan test).

Components of carbon storage	Thinned (t C ha^−1^)	Underplanted (t C ha^−1^)	Unthinned (t C ha^−1^)
Vegetation aboveground carbon storage			
* M. glyptostroboides *	95.91 (6.82)^a^	97.78 (9.43)^a^	60.22 (5.95)^b^
Understory wood plant	0.78 (0.15)^b^	4.63 (0.64)^a^	0.18 (0.03)^c^
Herbaceous plant	0.71 (0.11)^a^	0.23 (0.03)^b^	0.06 (0.01)^b^
Sum of aboveground	97.4 (6.27)^a^	102.64 (8.29)^a^	60.46 (5.17)^b^
Vegetation underground carbon storage			
* M. glyptostroboides *	25.71 (3.26)^a^	26.20 (2.98)^a^	16.21 (3.02)^b^
Understory wood plant	0.23 (0.04)^b^	2.53 (0.42)^a^	0.05 (0.01)^c^
Herbaceous plant	0.13 (0.01)^a^	0.04 (0.01)^b^	0.01 (0.00)^b^
Sum of underground	26.06 (1.36)^a^	28.77 (2.83)^a^	16.27 (1.68)^b^
Litterfall	2.14 (0.39)^ab^	2.54 (0.32)^a^	1.75 (0.08)^b^
Soil organic carbon	123.06 (9.71)^ab^	135.40 (7.95)^a^	118.84 (10.06)^b^
Total ecosystem carbon storage	248.66 (16.43)^a^	269.35 (21.51)^a^	197.32 (19.38)^b^

**Table 3:** Soil light fraction organic carbon content in thinned, underplanted, and unthinned stands (standard deviation in brackets); different letters in the same soil layer indicate there are significant differences among the three treatments (5% level, Duncan test), small letters denote the difference in LFOC, and capital letters denote the difference in LFOC/SOC.

Soil layer (cm)	Thinned		Underplanted		Unthinned	
	LFOC	LFOC/SOC	LFOC	LFOC/SOC	LFOC	LFOC/SOC
	(g kg^−1^)	(%)	(g kg^−1^)	(%)	(g kg^−1^)	(%)
0–15	7.06 (1.25)^a^	30.00 (3.15)^A^	6.85 (1.34)^a^	30.09 (3.46)^A^	5.21 (1.08)^b^	33.49 (3.06)^A^
15–30	2.53 (0.83)^a^	18.25 (2.34)^A^	2.64 (0.68)^a^	16.27 (2.12)^A^	2.14 (0.75)^a^	17.08 (2.40)^A^
30–50	0.42 (0.21)^a^	5.38 (1.64)^A^	0.51 (0.26)^a^	4.80 (1.35)^A^	0.45 (0.19)^a^	4.67 (1.28)^A^
50–100	0.08 (0.05)^a^	1.50 (0.58)^A^	0.06 (0.05)^a^	1.05 (0.41)^A^	0.05 (0.04)^a^	0.89 (0.52)^A^

**Table 4:** Correlation analysis between soil respiration and soil temperature or soil water content at the temporal scale.

Treatment		*r*	*P*
Thinned	CO_2_ versus soil temperature	0.856	0.035
	CO_2_ versus soil water content	0.832	0.078
Underplanted	CO_2_ versus soil temperature	0.845	0.021
	CO_2_ versus soil water content	0.909	0.047
Unthinned	CO_2_ versus soil temperature	0.901	0.015
	CO_2_ versus soil water content	0.972	0.034
